# TP53LNC-DB, the database of lncRNAs in the p53 signalling network

**DOI:** 10.1093/database/bay136

**Published:** 2019-01-08

**Authors:** Muhammad Riaz Khan, Ihtisham Bukhari, Ranjha Khan, Hafiz Muhammad Jafar Hussain, Mian Wu, Rick Francis Thorne, Jinming Li, Guangzhi Liu

**Affiliations:** 1 Translational Research Institute, Henan Provincial People’s Hospital, School of Medicine, Zhengzhou University, Zhengzhou, China; 2 Joint Centre for Human Reproduction and Genetics. Anhui Society for Cell Biology. School of Life Sciences, University of Science and Technology of China, Hefei, China

## Abstract

The TP53 gene product, p53, is a pleiotropic transcription factor induced by stress, which functions to promote cell cycle arrest, apoptosis and senescence. Genome-wide profiling has revealed an extensive system of long noncoding RNAs (lncRNAs) that is integral to the p53 signalling network. As a research tool, we implemented a public access database called TP53LNC-DB that annotates currently available information relating lncRNAs to p53 signalling in humans.

## Introduction

Long noncoding RNAs (lncRNAs) are broadly defined as RNA transcripts of >200 nucleotides with no protein-coding activity (1). Apart from translation, the biosynthesis and regulation of lncRNAs occur similarly to coding gene transcription with RNA polymerase II–mediated transcription, capping and polyadenylation, along with alternative splicing (2, 3). Notably, current estimates suggest that lncRNA genes may be nearly as abundant, if not more so, as their mRNA cousins (4). Indeed, there is growing interest in the function of specific lncRNAs in both physiological and pathological processes (5–7).

Among the body of work involving lncRNAs, there has been a strong association with the tumor suppressor p53 (8, 9). P53 is the protein product of the TP53 gene, and among a large group of genes defined as either tumor suppressors or oncogenes, TP53 is arguably the most notorious gene linked with the aetiology of cancer. This reputation comes from the observation that TP53 is rendered ineffective by mutation in ~50% of all cancer cases and possibly inactivated by a range of indirect mechanisms in others (10, 11). It has become well known that p53 directly or indirectly regulates a diverse array of cellular pathways (12, 13). Most notably, the p53 signalling network is essential for normal cell growth and genomic stability, working to impose cell cycle arrest or apoptosis in response to cellular stress, including but not limited to DNA damage and environmental challenges, together with responding to oncogenic changes in cells (14).

The cellular levels of p53 are tightly controlled, and these have been shown to be regulated by an array of transcriptional and post-transcriptional mechanisms (15, 16). For example, p53 levels are classically regulated by proteasomal degradation mediated through the E3 ubiquitin ligases MDM2, COP1 and Pirh2, among others (16–19). However, there is a growing body of evidence to establish links between lncRNAs and the p53 signalling network. In particular, a large number of lncRNAs appear to be implicated in cellular signalling pathways alongside of p53, functioning as regulators or effectors in the execution of downstream functions of p53 (20). For example, p53 transcriptionally upregulates the lncRNA TRINGS (Tp53-regulated inhibitor of necrosis under glucose starvation) to protect cancer cells from necrosis under conditions of glucose starvation (21). Similarly, the lncRNA PANDA is also upregulated by p53, working to regulate cell death genes downstream of p53 (22). Other lncRNAs have been shown to exert control over p53, accomplishing this through a variety of mechanisms. The lncRNA DINO binds directly to the p53 protein and promotes its stabilization (23), while MEG3 also impacts p53 gene expression through effects of MDM2 that prevent p53 degradation (24). Conversely, the lncRNA RoR represses p53 in response to DNA damage and has unique capability being as effector and regulator of p53, forming an autoregulatory feedback loop (25).

Recently, improved computational predictions together with more sensitive RNA sequencing (RNA-seq) technologies have helped to uncover the true extent of the lncRNA transcriptome (26, 27). The latter is important as the expression level of lncRNAs is often quite low compared with mRNAs (28). There are an increasing number of publications detailing the specific roles of individual lncRNAs across a spectrum of p53-regulated signalling pathways (8, 9, 29), most notably the dysregulation of lncRNAs in cancer and their contribution to disease pathophysiology (21, 30). Keeping in mind the growing importance of this research, we designed and implemented a comprehensive database ‘TP53LNC-DB’ providing the accumulated knowledge of human lncRNAs specifically involved or implicated in p53 signalling pathways.

## Data source implementation and contents of database

Articles published before 31 May 2018 were retrieved from PubMed using keyword searches [p53 AND long non-coding RNA], [p53 AND lncRNA] and [p53 AND lincRNA]. Abstracts were curated, and information was extracted as a manually annotated record of database fields including linked entries to PMIDs of the relevant articles (Table 1). Abstracts of the retrieved articles were read to confirm if each study was related to the p53 signalling pathway. Thereafter, high-confidence data were collected from these studies to appropriately match data fields required for entry into the database.

**Table 1:** Detailed field entry data for TP53LNC database

Field code	Entry variables	Description of criteria
P53LncRNA_ID	P53LNCxxxx	System generate unique identifier for TP53LNC database
LNCipedia_ID		Unique identifier assigned from the LncRNAdb project with link out service to LNCipedia.org entry
Ensembl_ID	ENSTxxxx	The transcript variant associated with the gene symbol with link out service to Ensemble
NONCODE_ID	NONHSATxxxx	The transcript variant associated with the gene symbol with link out service to NONCODE
Symbol		The lncRNAs identified by its common HNGC identifier along with available identifying information retrieved from each publication
Verification	*Predicted* OR *Verified* (If verified methods specified)	LncRNAs associated with p53-signalling either Predicted (no further experimental evidence available) or Verified (supported by additional experimentation)
Method	The experimental methods that were employed to identify the lncRNAs, such as microarray, RNA-seq, Tilling array, etc.	The experimental approach by which a particular or a large dataset(s) LncRNA related to p53 signalling was identified
p53_regul.	Up-regulated, Down-regulated, N/A	Up-regulated/ Down-regulated according to positive or negative regulation by p53. N/A if unknown
p53_pathway	Pathway(s) OR cellular process(es) such as DNA damage response (DDR), cell cycle, senescence, proliferation, etc.	Particular signalling pathway(s) or processes involved where lncRNAs are Verified
Tissue_Cells	Cell line (tissue source/name specified)	The specific cell(s) type OR tissue(s) (cancerous) in which lncRNA(s) are identified
PMID		Link out to PMIDs of the relevant article

**Figure 1:**
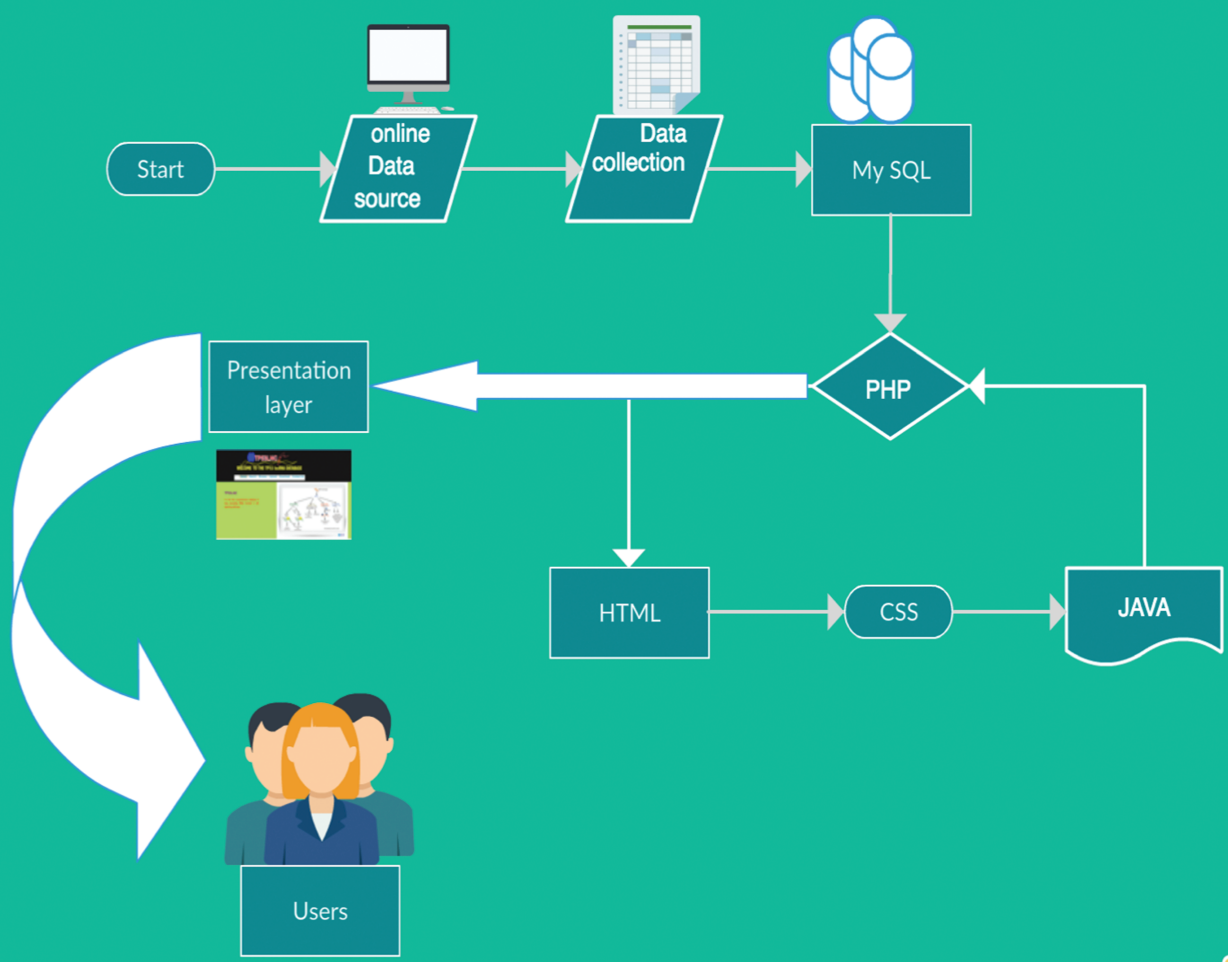
Layout of application architecture. The collected lncRNA in database information was integrated as PHP presentation layer combined with MySQL persistent storage. HTML/CSS and JavaScript interfaces were given for interpretation and navigation.

**Figure 2:**
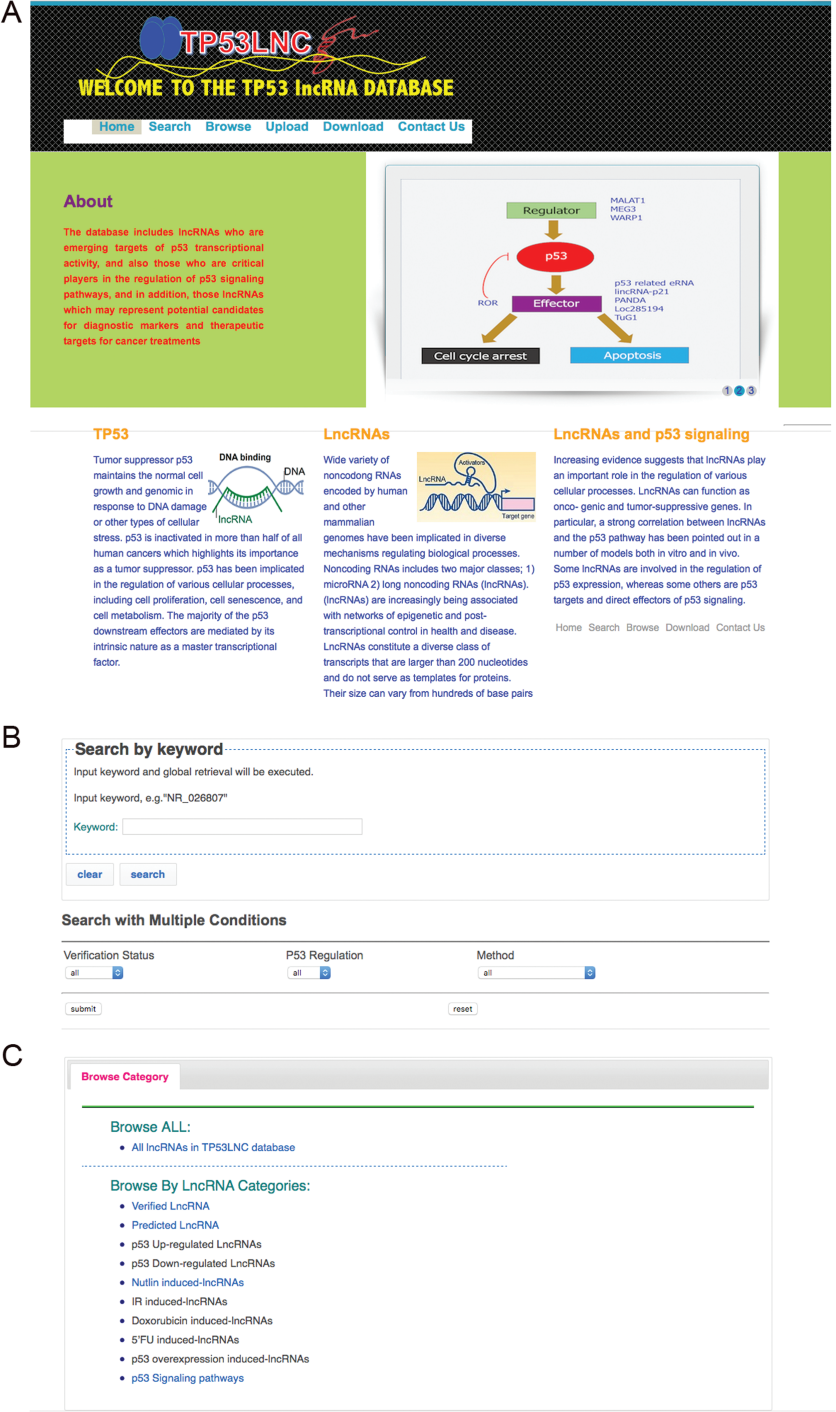
The web interface of TP53LNC-DB. (A) The homepage screen, (B) search screen and (C) preconfigured browsing options.

First, each lncRNA entered into database was assigned a unique identifying number (P53LncRNA_ID). Entries derived from high-throughput approaches including RNA-seq, deep sequencing and microarrays but not having been verified by secondary approaches were classified as ‘Predicted’ in the ‘Verification’ field. If secondary approaches were used to confirm the lncRNA-p53 association, then these lncRNAs were classified as verified and the methods used were recorded in the ‘Verification’ field. For example, Leveille *et al.* (31) globally mapped p53-regulated enhancers by treating MCF-7 cells with the p53 activator nutlin3a. After RNA-seq analysis and identification of differentially induced lncRNAs, the nutlin3a-responsive lncRNA LED was confirmed by several experimental approaches including qPCR, ChIP and Northern. Thus LED is listed as a ‘Verified’ lncRNA, while other p53-regulated lncRNAs not investigated are listed as ‘Predicted’. Other examples of ‘Verified’ entries include TRINGS (21) and GUARDIN involved in maintaining genomic stability (30). Both lncRNAs were first identified by microarray analysis using H1299 cells carrying an inducible wild-type p53 expression system. After initial screening, both TRINGS and GUARDIN were confirmed to be p53-responsive lncRNAs using a variety of secondary approaches to reveal their function. The ‘Method’ field captures further information regarding the primary screening approach and other methods used to identify the lncRNAs.

As lncRNAs can be either positively or negatively impacted by p53, their regulatory status is recorded in the ‘p53_regul.’ field in the database as ‘Up regulated’ or ‘Down regulated’. Where the status is undetermined or the lncRNA expression unaffected by p53, e.g. as part of the TP53 pathway but not regulated by it, the entry is marked not applicable (NA) for this category field. Additional information regarding the function of each lncRNA in p53 signalling is recorded in the ‘p53_pathway field’, such as DNA-damage response, proliferation and apoptosis for the entry for the lncRNA GUARDIN. In some instances, the identified lncRNAs act as regulators of TP53; for example, HSUP1 is indicated to destabilize p53, this information also being captured within the ‘p53_pathway field’. Lastly, the corresponding experimental approaches used together with cells and/or tissue data were captured for the ‘p53_pathway’ and ‘Tissue_Cells’ fields, respectively.

In addition to the annotated data fields derived from PubMed, the lncRNA gene names or transcript names obtained through PubMed were used to retrieve relevant transcript or gene IDs from other data sources. The database includes web links to LNCipedia (https://lncipedia.org/), Ensembl (https://asia.ensembl.org/index.html) and NONCODE (http://www.noncode.org/index.php) to provide detailed lncRNA transcript information. For example, the lncRNA HOTAIR has links to the LNCipedia (HOTAIR), Ensemble (ENST00000424518) and NONCODE (NONHSAT028510) databases. Where particular transcript identifiers are not available in the corresponding databases, the entry is left blank. The ‘Symbol’ field contains the common gene symbol from HUGO Gene Nomenclature Committee (HGNC), if available, together with other alias information or identifiers obtained through the cited paper. It should be noted that several lncRNA entries are repeated in the database with different TP3LNC IDs, resulting from the same gene being identified through different studies. For example, searching for the lncRNA HOTAIR returns entries related to five studies (P53LNC0036 P53LNC0066, P53LNC0140, P53LNC1458, P53LNC2317) the information related to different transcripts as well as listing the specific attributes of each report. Leaving the information in this discrete form ensures the information from separate studies is easier to interpret.

## Development of database

The application architecture consists of a PHP presentation layer and MySQL persistent storage (Figure 1). Combined HTML/CSS and JavaScript enable interfaces that are easy to interpret and navigate. TP53LNC-DB is supported by main standards-compliant web browsers including Firefox, Google Chrome, Internet Explorer and Safari.

**Table 2:** Summary of preconfigured search terms for browsing TP53LNC-DB entries

Grouping	Pre-configured database queries	Search term employed	Total entries/ group
Group I Verification status	Verified lncRNAs	227	4851
	Predicted lncRNAs	4624	
Group II Regulation status	p53 Up-regulated lncRNAs	2680	4094
	p53 Down-regulated lncRNAs	1414	
Group III p53-lncRNA inducing agents and others	Nutlin-induced lncRNAs	769	4526
	IR-induced lncRNA	785	
	Doxorubicin-induced lncRNAs	25	
	5’-FU–induced lncRNAs	25	
	p53 overexpression–induced lncRNAs	2804	
	Others	118	

## Data query search and browsing

The front end of TP53LNC-DB is a simple user-friendly web interface (Figure 2A) with access to information provided by text-based search (Figure 2B). Searches are configured to match either full or partial query terms. For example, searching for the keyword ‘DINO’ will retrieve the entry for lncRNA DINO, whereas a partial search term such as ‘DIN’ will retrieve the entries for DINO and GUARDIN. Multi-conditional search options are also configured for key parameters, whether the lncRNA(s) are experimentally verified or predicted, the conditional status of p53 being up-regulated or down-regulated and the implemented experimental approach. Total database entries can also be viewed in the browsing function along with the display of preconfigured search queries aligned with different aspects of p53 biology (Figure 2C; Table 2).

## Discussion

The knowledgebase around lncRNAs is considerable less than that of protein-coding genes. Notably, however, there are other aspects of lncRNA biology that impede research efforts (26). Annotating the lncRNA transcriptome is inherently difficult for several reasons; their relative low expression causes lncRNA transcripts to be under-sampled in high-throughput RNA-seq, and they lack identifiable features such as open reading frames (ORFs). Moreover, the weak or absent conservation of lncRNAs necessitates a heavy reliance on empirical studies. On the latter basis together with our own research interests in p53, we initiated the TP53LNC-DB project.

Many databases have already been developed to store lncRNA-associated information (8, 32–34); however, an lncRNA database dedicated to a singular gene such as p53 signalling has never been previously implemented. At time of publication, there were 4851 unique lncRNA entries correlated with p53-related signalling pathways in the database, a figure that clearly speaks to the importance of this emerging aspect of p53 biology. However, less than 5% of these entries have been experimentally verified either *in vitro* or *in vivo* (Table 2). We anticipate the database will help de-convolve this complexity of information around p53 and lncRNAs and prove a useful resource to the research community.

## Notes

**Database URL**: http://www.trihpph.net/TP53LNC/index.php
